# Distance to large rivers affects fish diversity patterns in highly dynamic streams of Central Amazonia

**DOI:** 10.1371/journal.pone.0223880

**Published:** 2019-10-17

**Authors:** Lis F. Stegmann, Rafael P. Leitão, Jansen Zuanon, William E. Magnusson

**Affiliations:** 1 Programa de Pós-Graduação em Ecologia, Instituto Nacional de Pesquisas da Amazônia, Manaus, Amazonas, Brazil; 2 Departamento de Genética, Ecologia e Evolução, Universidade Federal de Minas Gerais, Belo Horizonte, Minas Gerais, Brazil; 3 Coordenação de Biodiversidade, Instituto Nacional de Pesquisas da Amazônia, Manaus, Amazonas, Brazil; University of Arkansas Fayetteville, UNITED STATES

## Abstract

Longitudinal-zonation hypotheses generally predict gradual changes in fish composition from upstream to downstream due to changes in habitat conditions, but largely disregard downstream effects on upstream segments. Floodplains of large rivers represent areas of high connectivity during flood periods and can act as stable refuges in dry seasons, which may attenuate deterministic constraints imposed by local conditions on fish assemblages in surrounding habitats. In this study, we investigated the effects of proximity to large rivers on taxonomic- and functional-diversity patterns of stream-fish assemblages in an extensive region of Central Amazonia. We sampled 31 headwater-stream reaches in nine catchments in the Purus and Madeira Rivers interfluve between December 2014 and March 2015. Ninety seven fish species from seven orders and 19 families were captured. The results indicate that distance to large rivers is more important than distance among sites and local conditions in explaining functional and taxonomic diversity of stream-fish assemblages at large spatial scales. We also found a decrease in taxonomic and functional richness towards headwaters, mainly related to the loss of benthic and sedentary species along the distance gradient. These species may be favored by the proximity to refuge areas and high resource availability near the floodplain. In contrast, upstream assemblages were mainly occupied by small-sized, nektonic species with higher dispersal capacity, highly dependent of allochthonous resources. Downstream effects could be detected for many kilometers upstream in hydrographic catchments and this reinforces the crucial role of connectivity between fluvial habitats in maintenance of stream-fish diversity patterns in the region.

## Introduction

Longitudinal fish zonation occurs when assemblages undergo changes along the linear gradient that connects upstream and downstream portions of a river network [1]. In general, increased availability and heterogeneity of habitats that occur along the longitudinal axis facilitate coexistence of species and the gradual accumulation of species in the downstream direction [1–5]. However, this perspective disregards the effects of downstream habitats on upstream segments, which may limit deterministic constraints imposed by local conditions [6–9], especially for organisms with high dispersal abilities, such as fish that can transit between habitat patches. Therefore, freshwater assemblages are not determined solely by unidirectional effects [6,10–14] and physical disturbances and spatial arrangement are important elements maintaining some attributes of habitats and ecosystem functions [8,15].

Proximity to large rivers increases dispersal opportunities between habitats and availability of allochthonous resources, amplifying the role of mass-effects and source-sink dynamics in local and regional fish assembly [6,7,13,16]. Large-river floodplains provide greater environmental heterogeneity during high-water seasons, favoring lateral migrations and reducing competitive pressures [17]. The severity of hydrological disturbances in the dry season may depend on the spatial position of a stream within the drainage network and assemblages well connected to the floodplain should receive more migrants that buffer against demographic stochasticity and allow fast recovery from disturbance events [8,18]. Several studies have shown that low-order streams flowing directly into large rivers (i.e. adventitious streams) may have higher numbers of species than similar-sized streams located further up the network due to migration from the larger rivers [6,7,19,20]. Less well known are the effects of this connectivity on fish species that do not undertake large migrations, but are favored by lateral connectivity with marginal environments that allows them to avoid competitive pressures from species in the stream channel [21,22].

In a functional perspective, the river-continuum hypothesis predicts that headwater systems should be primarily occupied by small-sized and nektonic fish species mainly dependent on allochthonous resources, while downstream systems should harbor larger species predominantly supported by autochthonous production, especially for benthic fish guilds [5,12,23]. However, increased connectivity with large rivers can reduce effects of local filters on assemblages and increase opportunities for opportunistic migration of species that typically do not reside in low-order stream, thereby increasing local functional richness [10,20]. Fish species also differ in their abilities to move through connecting waterways [24], and in strongly dynamic systems, where fish need to migrate following the water level, dispersal capacity can be a determining factor both to reach the refuges in the dry season and to recolonize new environments when the flow is reestablished [25]. Changes in functional structure of fish assemblages may facilitate the disentangling of effects of disturbances and habitat isolation as well as predicting potential changes in key ecological processes across the landscape [26,27].

Beta-diversity indices are useful tools to infer changes in species composition between sites and to identify the ecological gradients that structure these changes. Several methods have been proposed to partition the two main components of dissimilarity between habitats: species turnover and richness differences/nestedness [28,29]. Species turnover, or replacement, refers to simultaneous gains and losses of species in the landscape and can be linked to local conditions when species can move to favorable environments [30], or to stochasticity and drift when habitats are isolated and differences are accumulated across the landscape [31–33]. Richness differences or nestedness can reflect changes in resource availability or the effects of spatial filters [16,34]. Identifying the contribution of each dissimilarity component in different portions of a landscape allows better predictions about the ecological process acting on biological assemblages.

In this study, we investigate how proximity to large rivers determines the taxonomic and functional composition of fish assemblages in headwater systems in an extensive region of Central Amazonia. The region is characterized by a highly dynamic landscape where headwater streams can dry completely during drought months, and/or receive inputs from floodplains in the high-water season, depending on their distance to large rivers. We hypothesized that fish assemblages in similar-sized streams would differ in taxonomic and functional richness as a function of distance to large-river floodplain systems. This pattern should be determined mainly by the greater occurrence of species that benefit from the proximity to refuge areas and from the higher productivity of the floodplain, such as detritivorous benthic and less dispersive species. In contrast, fish assemblages in streams further from large rivers should be dominated by species with better dispersal abilities and sustained by allochthonous food sources. We expected distance to the large rivers to be an important factor structuring stream-fish assemblages, and that streams similarly positioned in the drainage network would share a greater number of species and functional traits, regardless of local conditions and watercourse-distances among streams.

## Methods

### Study area

**Fig 1:**
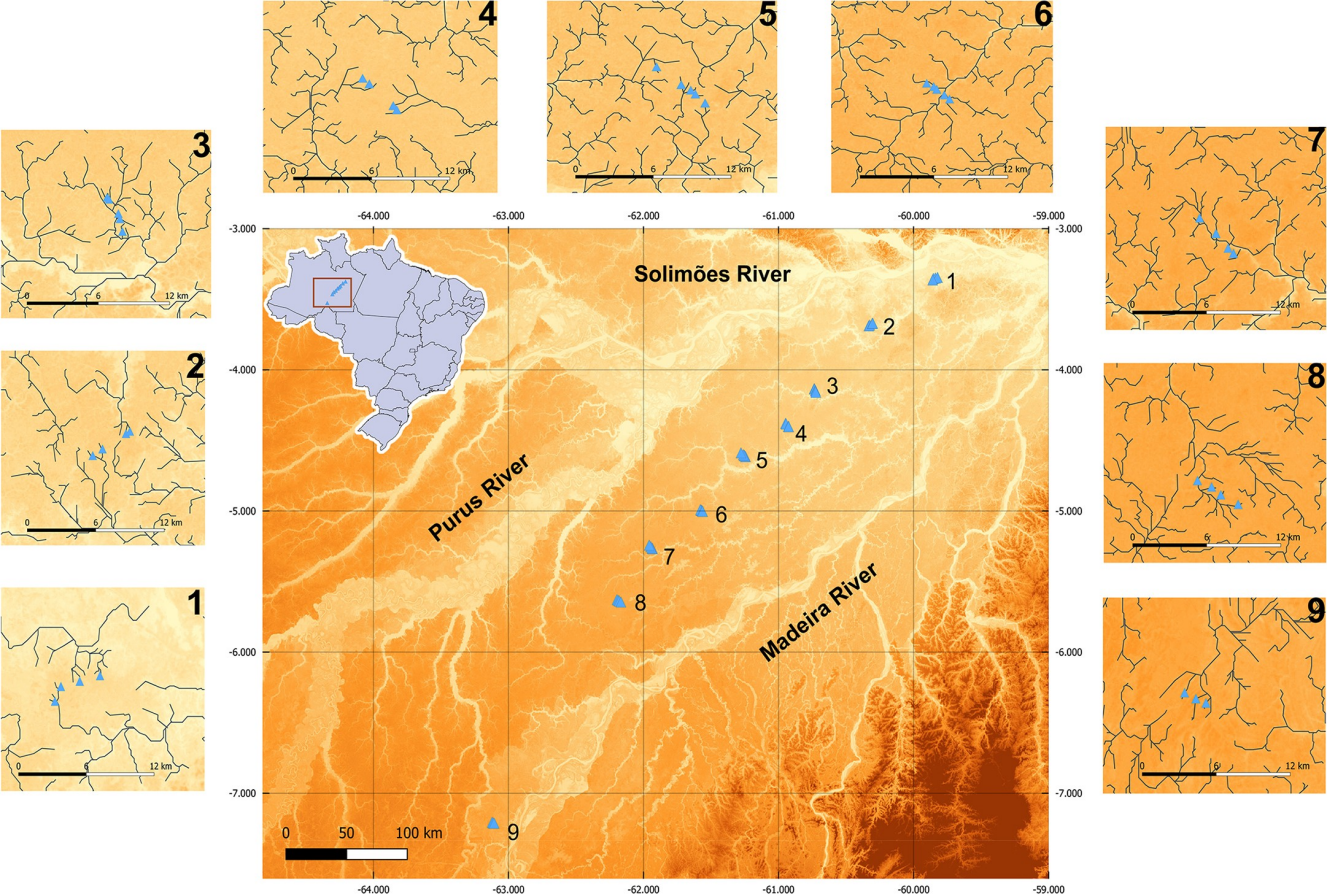
Elevation model of the study area showing the Purus-Madeira interfluve in Central Amazonia, Brazil. Blue triangles correspond to the 31 sampled streams, which are distributed in nine catchments (numbers). Smaller figures show each sampled catchment in detail. Darker shades indicate higher altitudes (range: 12 to 371 meters above sea level).

Sampling was undertaken along 700 km of the Madeira-Purus interfluve, in southwestern Amazonas State, Brazil. The region is covered by dense rainforest with mean annual precipitation varying from 2000 to 2400 mm [35] (Fig 1). Predominant soil texture in the region is silt to fine sand and relief varies from flat to gently undulating, which results in poorly drained soils [35]. The combination of flat relief and high annual flood pulses of the Purus, Madeira and Amazon Rivers, which usually exceed 10 m [36], creates marginal lakes and temporally inundated systems during the wet season. However, the shallow water-table does not sustain surface flow in headwaters through consecutive drought months when the rain stops, causing smaller streams to dry completely for several weeks, or for free water to persist only in isolated pools along the stream bed. This hydrologic fluctuation determines a highly dynamic landscape, especially in headwater systems, which experience drought and flood conditions within short periods of time.

We sampled 31 headwater-stream reaches (50-m long reaches in 1^st^ to 3^rd^ order streams) in nine catchments along the Madeira-Purus interfluve that drain to the Madeira or Amazon Rivers. The dense network is mainly non-hierarchical and the sub-basins can be short and flow directly to large rivers or be extensive and receive little influence from the floodplain of large rivers. Sampling was carried out in the first months of the rainy season, between December 2014 and March 2015, when the streams returned to normal flow and the water level remained stable for months, allowing rapid recolonization of formerly dry stream channels. During the inundation phase, small streams overflow and vast areas are flooded, including parts of the road, making access difficult. These limitations make it hard to obtain more accurate information about the extent of flooding in terrestrial habitats. All stream segments sampled are permanent sampling plots of the Research Program in Biodiversity (PPBio) field-site network (http://ppbio.inpa.gov.br/sitios/br319).

### Sampling

#### Stream-habitat variables

In each stream reach, we measured water temperature, electrical conductivity, dissolved oxygen and pH with a YSI Pro 1030^®^ multiparameter probe. We also measured channel width, depth, substrate type and flow velocity in four transects perpendicular to the stream channel, separated from each other by 12.5m. Depth and substrate type were measured in nine equidistant points along each transect across the stream channel. Substrate was classified in one of six categories: sand, clay, trunk (wood with diameter >10 cm), coarse litter (leaves and small branches), fine litter (organic silt) or roots (fine roots from riparian vegetation). Flow velocity was estimated as the average time that a floating object took to travel 1 m, replicated three times. For statistical analyses, we use the average values of channel width, depth, flow velocity and the percentage of each substrate type of each stream reach. For details regarding the environmental-sampling protocol see [37].

#### Fish sampling

In each stream, the 50-m long reach was isolated with block nets (5mm mesh) and fish were caught using seines and hand nets. Although 100 to 150-m stream sections have been used for stream-fish studies, the use of 50-m long stream reaches allows robust inferences regarding both taxonomic and functional fish diversity in such water bodies (see the sensitivity analyses provided by the electronic supplementary material S5 in [26]). Sampling larger segments of first-order streams reduces the precision of predictor variables, such as stream depth, because streams may become very small or disappear over distances of 100m [38], and the extra precision in description of fish assemblages does not compensate for the reduction in replication due to time constraints for sampling in this area of difficult access. Furthermore, repeated sampling over nearly 20 years in 50-m reaches of upland forest streams have revealed a high stability and predictability of local fish assemblages, that support the use of such stream-reach size for ecological analyses (JZ pers. observations in Reserva Ducke near Manaus, Amazonas state, Brazil, between 2001 and 2019).

Fish were collected only during the day and collection effort in each section was standardized by using two collectors over two hours. The specimens were euthanized in clove-oil solution, which decreases fish neurosensory functions by acting upon the nervous systems. The specimens were fixed in 10% formalin and preserved in 70% ethanol. Fishes were sorted, counted and identified to species level in the laboratory using dichotomous keys and specialized literature, such as [39], [40], [41] and [42]. All collected specimens were deposited in the fish collection of the Instituto Nacional de Pesquisas da Amazônia (INPA). The sampling procedures were approved by INPA's Institutional Committee for Ethics in the Study of Animal (Comissão de Ética no Uso de Animais #052/2012) and a research license was provided by the Brazilian Institute of the Environment and Renewable Natural Resources (Instituto Brasileiro do Meio Ambiente e dos Recursos Naturais Renováveis, SISBIO#10.199–1).

#### Functional traits

Each fish species was functionally characterized with respect to locomotor ability, habitat use and food intake. Body mass and morphometric measurements were taken from 3–10 adult individuals per species and then combined in 13 ecomorphological traits (see S1 Text and S2 Table for details).

The functional traits were used to calculate a pairwise functional distance between species using Euclidian distance. We then ran a principal components analysis (PCA) on the functional-distance matrix to obtain the coordinates of the species in multidimensional space. To define the number of dimensions that best describes the functional space, we used the framework developed by [43], which analyzes the congruence between the initial functional distance (Euclidian distance matrix) and the distance between each pair of species in the functional space. This indicated that the first four PCA axes used to build the functional space closely reflected the initial functional dissimilarity between species (mean squared deviation between functional standardized distance and initial distance <0.001). Therefore, we kept the first four axes to describe the functional space, representing 76.1% of the inertia. We had to exclude two assemblages with less than four species due to computational requirements (i.e. higher number of species than PCA axes), as proposed by [44].

To evaluate how functional space varies between fish-assemblages, we computed the functional-richness index (FRic), which represents the amount of functional space filled by all species within the assemblage, indicating the range of trait combinations or niche occupation [41]. To investigate how functional-trait values vary in each assemblage, we calculated the community-weighted mean (CWM hereafter) of each trait, which represents the average value of each functional trait in the assemblage based on the occurrence of the species and their relative abundances. The indices were calculated using the script “FD” developed by [45] using R software [46].

#### Beta-diversity indices

We calculated the variation in taxonomic species composition (Taxonomic Beta Diversity, hereafter Tβsør) among the 31 stream reaches by means of pairwise comparisons using Sørensen-based index developed by [47] for use with presence/absence data. To evaluate the role of the different ecological processes on spatial variation among fish assemblages, we decomposed the Sørensen-based index in relativized richness difference (species gains and losses, Tβrich) and relativized species replacement (Tβrepl). We chose the Tβrich instead of the nestedness component [48] because the first incorporates several compositional differences attributable to richness and not only those due to nestedness [29]. The beta-diversity indices were computed using the script proposed by [29] using R software.

Variation in functional diversity between stream reaches (hereafter Fβsør) was calculated from pair-wise intersections between convex hulls in a multidimensional functional space created with the first four PCA axes using the Sørensen dissimilarity index. Fβsør was decomposed into functional turnover (Fβsim) and functional nestedness (Fβnes), following the framework proposed by [44]. Functional turnover is measured as the amount of overlap in functional space between two assemblages, and the functional nestedness represents the amount of functional space of one assemblage that is filled by another [44]. The indices were calculated from the “betapart” package developed by [49] using R software.

#### Geographical and abiotic predictors

To test the effect of distance to large rivers on diversity patterns, we measured the distance between each stream reach and the nearest large river (Madeira or Amazon Rivers) following the watercourse. The distances were calculated by summing the lengths of segments between each stream reach and the nearest large river in a GIS hydrological framework developed by [50] using QGIS 2.6 software. The distance measurements for each stream reach are given in S1 Table. We used these data to create a dissimilarity matrix using Euclidian distances (differences in distance to large rivers).

To evaluate the effect of watercourse distance on taxonomic and functional patterns, we calculated the network distance between each pair of stream reaches using the QNEAT3 extension for QGIS Network Analysis in the GIS hydrological framework developed by [50]. We also calculated the linear distance between sites since the flat relief of the study area permits the overflow of small streams during flood seasons, possibly connecting them through the floodplain environment and allowing dispersal outside the main stream courses. The two distance matrices were highly correlated (Mantel statistic r = 0.89) so we used only watercourse distance as it represents a more usual measure in riverine landscape studies. To describe the differences in the local conditions of the streams, we calculated a dissimilarity matrix with Euclidean distances on standardized abiotic variables.

#### Statistical analyses

All statistical analyses were carried out using R software [46]. The relationships between the components of Tβsør and Fβsør and the predictor variables (difference in distance to larger rivers, watercourse distance and dissimilarity in local conditions) were assessed by matrix correlation using Mantel tests. Since the matrices of difference in distance to large rivers and watercourse distance are correlated (Mantel R = 0.59; P = 0.001), we used a partial Mantel correlation to control possible confounding effects and to assess the individual contribution of each matrix. This statistic computes the degree of relationship between assemblage dissimilarities and each predictor matrix while controlling for the effect a third potentially confounding variable. This is achieved by regressing each matrix on the third one and then computing a Mantel test with the residuals of the regressions [51]. This does not fully control the possible effects of spatial autocorrelation, but rather dissociates the effects of watercourse distance from the effects of differences in distance to large rivers on assemblage dissimilarities. We also used a partial mantel test to control the effects of local conditions on beta diversity indices. Mantel and partial Mantel tests were calculated using the “vegan” package [46].

Similarities within catchments could cause autocorrelation, so we also provide a partial Mantel test to examine the relationships between the beta diversity components and the predictor variables, including a catchment identity matrix as confounding factor to ensure that significant correlations are not determined by autocorrelation effects due to greater similarities within catchments. The catchment identity matrix consists of a dissimilarity matrix where pairwise comparison of stream reaches from the same catchment had value equal to zero and comparisons between streams of different catchment have had a value equal to one. The analysis results are given in S2 Text.

To evaluate how local conditions interact with distance to large rivers to determine functional characteristics of stream-fish assemblages, we summarized the effects of local conditions with a Principal Component Analyses (PCA) for the standardized abiotic variables and retained the first axis as a descriptive variable, which represented 52% of variation in local conditions. The loading values for each variable are given in S1 Table. Then we used the first PCA axis and the distance to large rivers as fixed effects in a linear mixed-effect model (LMM) for Functional Richness (Fric), taxonomic richness and all CWM values of each assemblage. Catchment was included in the model as a random variable to account for the nested design (stream within catchment). LMM analyses were conducted with the package “lme4” and the marginal and conditional R^2^ were calculated using the package “MuMIn” [46]. We also calculated the Moran’s *I* values using the residuals of linear mixed-models to evaluated if the significance of the relationship that we found is affected by spatial autocorrelation and the results are given in S2 Text.

## Results

**Fig 2:**
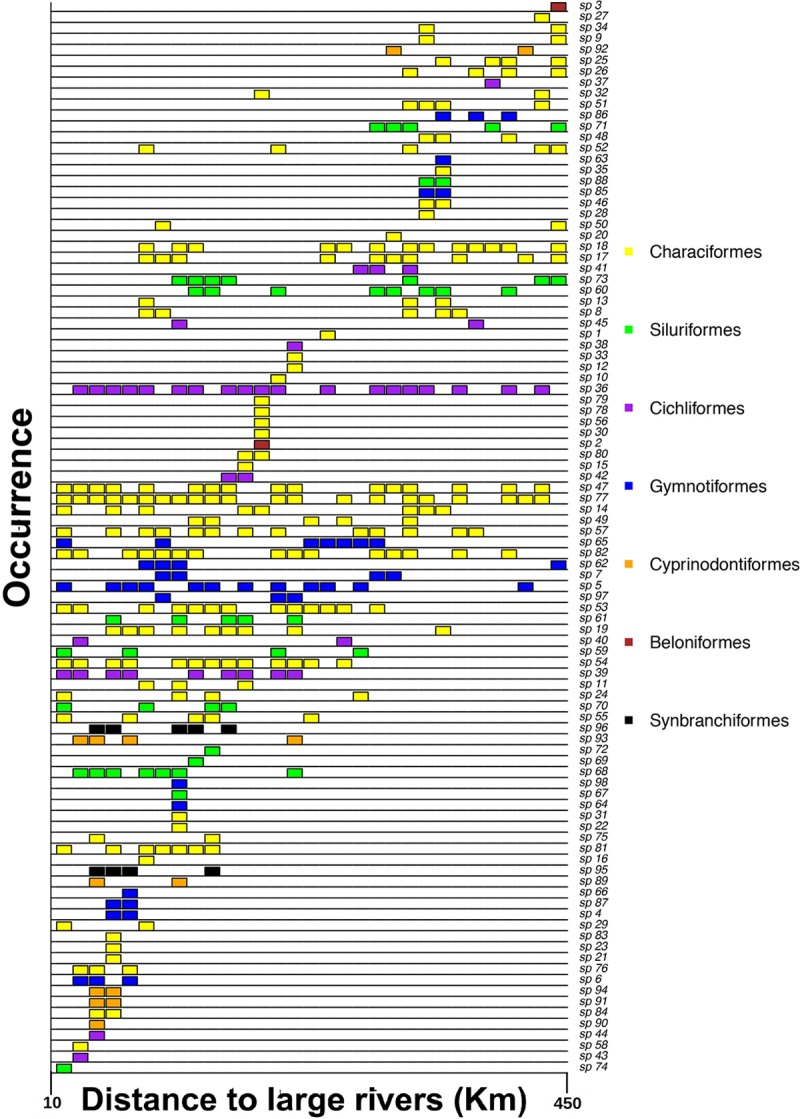
Fish species occurrence based on presence/absence data in 31 stream reaches ordered by the gradient of distance to large rivers. Different colors represent different fish orders. See S2 Table for full species names.

A total of 97 fish species from seven orders, 19 families and 57 genera were captured. Characiformes was the richest order with 52 species, followed by Siluriformes (14 species) and Gymnotiformes (12 species); the remaining 19 species were from four orders (Fig 2; S2 Table for details). Taxonomic richness ranged from 4 to 24 species per stream reach and 33 species were collected only once. Only four species were collected in more than 50% of streams (*Copella callolepis*, *Apistogramma agassizii*, *Crenuchus spilurus* and *Hemigrammus melanochrous*, in decreasing order of participation). Most species found in the streams were small-sized fishes typical of headwater systems and almost 75% of species had adult standard length less than 15 cm.

**Fig 3:**
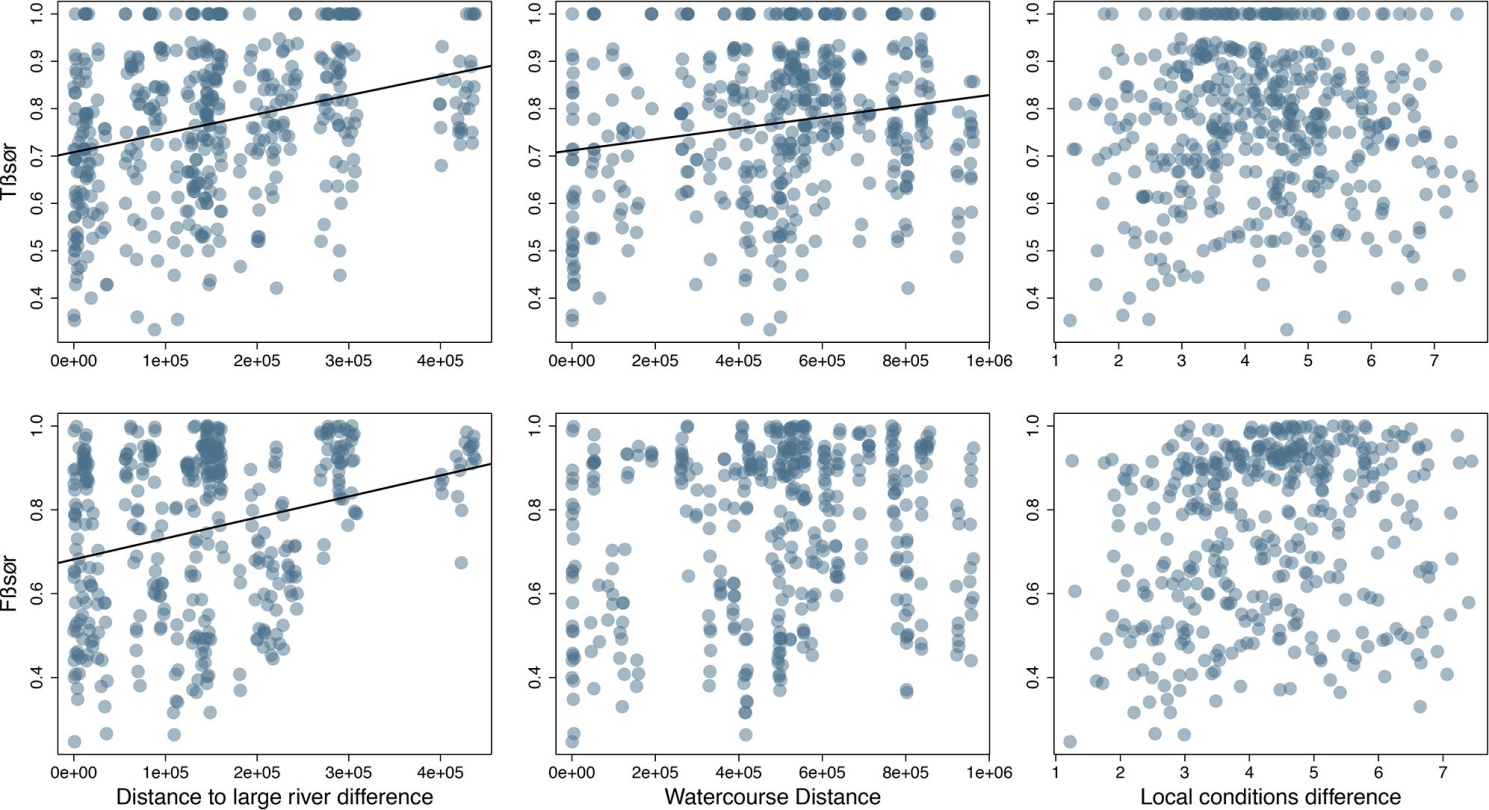
Relationships between taxonomic (Tβsør) and functional (Fβsør) beta diversity and difference in distance to large rivers, watercourse distance and local-condition differences. Fitted lines indicate probabilities <0.05.

We found a significant positive correlation between taxonomic beta diversity (Tβsør) and distance to large river, mainly associated with the richness component (Tβrich) (Fig 3). The partial Mantel tests indicated that this association was maintained even after controlling for the effect of watercourse distance and local abiotic effects (Table 1) and after controlling the effects of catchment identity (S2 Text). This indicates that streams similarly connected to floodplain systems are also similar in fish-assemblage composition, regardless of watercourse distance. There also appeared to be an effect of watercourse distance on richness differences (Tβrich), but this association was not statistically significant after controlling for the effects of distance to large rivers (Table 1).

**Table 1:** Summary of the Mantel correlations (*r* values) from Mantel tests and Partial-Mantel tests between functional and taxonomic beta-diversity of stream-fish assemblages and predictor variables.

		Distance to large rivers difference	Watercourse distance	Local conditions dissimilarity
Simple Mantel	Tβsør (A)	0.293**	0.187*	0.005
	Tβrich	0.335**	0.236*	0.029
	Tβrepl	-0.060	-0.06	-0.022
Partial Mantel	Tβsør (B)	-	0.016	-0.049
	Tβrich	-	0.048	-0.030
	Tβrepl	-	-0.027	-0.012
	Tβsør (C)	0.230**	-	-0.054
	Tβrich	0.249*	-	-0.039
	Tβrepl	-0.034	-	-0.008
	Tβsør (D)	0.297**	0.200*	-
	Tβrich	0.335**	0.242*	-
	Tβrepl	-0.060	-0.055	-
Simple Mantel	Fβsør (E)	0.222*	0.057	0.207
	Fβnes	0.044	0.027	-0.061
	Fβsim	0.088	0.009	0.171
Partial Mantel	Fβsør (F)	-	-0.085	0.168
	Fβnes	-	0.002	-0.072
	Fβsim	-	-0.049	0.159
	Fβsør (G)	0.230*	-	0.183
	Fβnes	0.035	-	-0.078
	Fβsim	0.101	-	0.201
	Fβsør (H)	0.186*	-0.029	-
	Fβnes	0.059	0.057	-
	Fβsim	0.053	-0.066	-

(A) and (E): Simple Mantel test. (B) and (F): Partial Mantel tests controlling the effects of differences in distance to large river. (C) and (G): Partial Mantel tests controlling the effects of watercourse distance. (D) and (H) Partial Mantel tests controlling the effects of dissimilarity in local conditions. Bold values correspond to statistically-significant correlations.

*p<0.05

**p<0.005.

Few species occurred continuously along the connection gradient to large rivers, indicating a strong pattern of substitution between catchments (Fig 2), and the beta-diversity analysis indicated that the variability in fish assemblages was more a result of species substitution (Tβrepl = 67% of total beta diversity) than loss or gain of species (Tβrich = 33%). However, we found no clear relationship between the turnover component (Tβrepl) and any predictor variables (Table 1). We also found no significant correlation between the taxonomic beta-diversity index and differences in local conditions.

**Fig 4:**
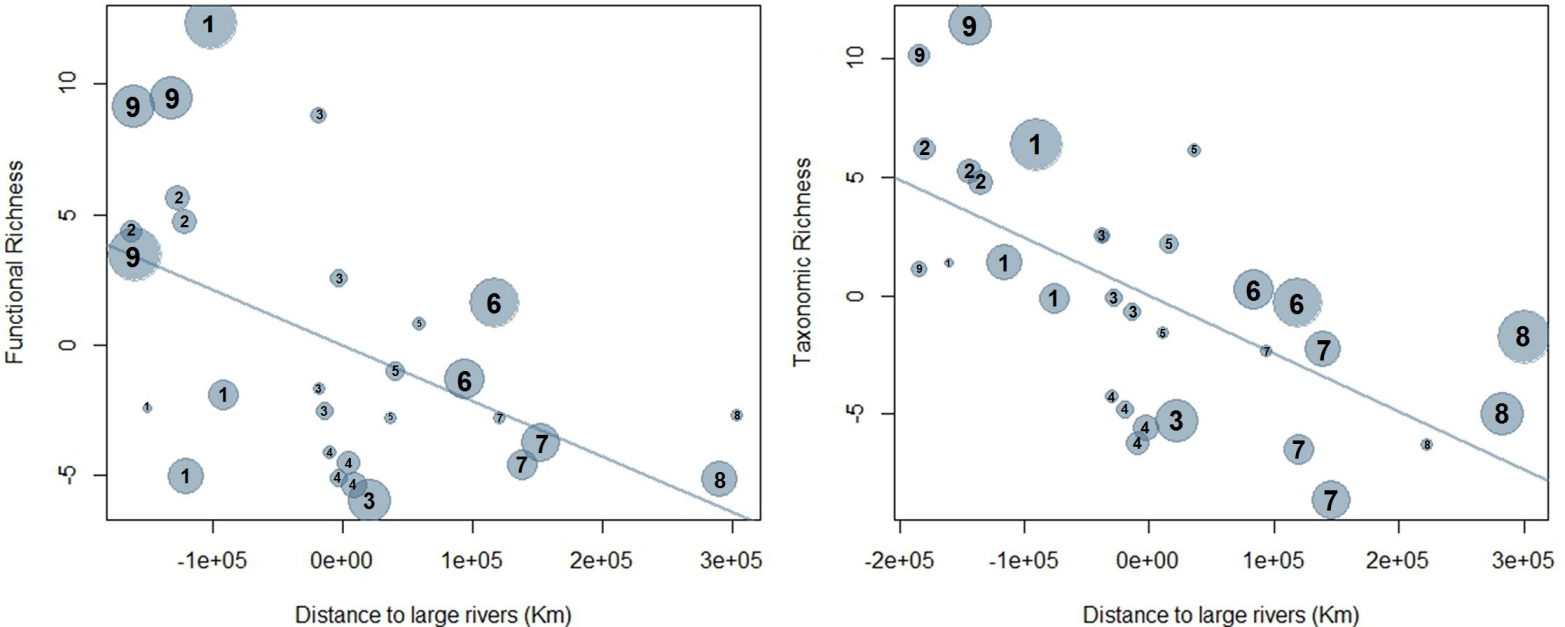
Partial regression derived from the linear mixed-effects models (LMM) investigating the effects of distance to large rivers on the functional and taxonomic richness of local stream-fish assemblages. Distance to large rivers ranged from 10 to 449 km. Circle size indicates the mean width of each stream reach and numbers represents the catchments (S1 Table for details).

In accordance with the Mantel tests, the LMM results indicated that distance to large rivers had a negative effect on richness of stream-fish assemblages (Table 2; Fig 4). The histogram of species frequency (Fig 2) shows a clear decrease in the number of species along the connection gradient to large rivers and a near disappearance of species of Synbranchiformes and Cyprinodontiformes in the upstream reaches.

**Table 2:** Probability associated with each predictor variable in the linear mixed-effects models (LMM) for CWM values and functional and taxonomic richness as functions of the distance to large rivers and local abiotic conditions (1^st^ PCA axis).

	Distance to large rivers	Abiotic conditions (1^st^ PCA)	R^2^ marg	R^2^ cond
Taxonomic Richness	**0.008**	0.207	0.419	0.674
Functional Richness	**0.010**	0.34	0.283	0.409
LogM	**0.048**	0.42	0.229	0.61
Osh	**0.003**	0.3	0.268	0.268
Ops	0.295	**0.001**	0.308	0.574
Edst	**0.026**	0.58	0.167	0.189
Eps	**0.024**	0.27	0.223	0.384
Bsh	0.880	0.35	0.036	0.157
Bsf	**0.021**	0.32	0.305	0.684
PFps	0.055	0.088	0.188	0.283
Pfar	**0.010**	0.79	0.226	0.304
CPt	0.081	0.4	0.172	0.515
CFar	0.170	0.76	0.1	0.281
Frt	0.220	0.38	0.061	0.061
Fsf	0.990	0.21	0.056	0.39

Catchment was considered a random effect in all models. Marginal R^2^ (R^2^ marg) values are for the models adjusted only considering fixed effects, and the conditional R^2^ (R^2^ cond) corresponds to the full model, including the random effects. Bold values correspond to p<0.05. LogM = log (body mass+1); Osh = Oral-gape shape; Ops = Oral-gape position; Edst = Eye size; Eps = Eye position; Bsh = Body transversal shape; Bsf = Body transversal surface; PFps = Pectoral-fin position; PFar = Aspect ratio of the pectoral fin; CPt = Caudal-peduncle throttling; Frt = Fin-surface ratio; Fsf = Fin-surface to body-size ratio; Cfar = Aspect ratio of the caudal fin.

There was a significant correlation between the functional beta-diversity index (Fβsør) and difference in distance to large rivers (Table 1), indicating that stream position influences the functional similarity patterns of the local fish assemblages. However, the difference in functional composition was not significantly related to either nestedness (Fβnes) or turnover (Fsim) components. Despite the lack of relationship between Fβnes and distance to large rivers, we found a decrease in functional richness in the upstream direction, indicated by the LMM results (Table 2; Fig 4), and there was no statistically-significant spatial autocorrelation in the LMM residuals (S2 Text). This indicates that the functional space, besides decreasing in upstream direction, is also changing along the gradient, thus preventing assemblages with greater functional richness completely filling the functional space of others and determining functional nested patterns. We found no relationship between local conditions and taxonomic or functional richness (Table 2).

**Fig 5:**
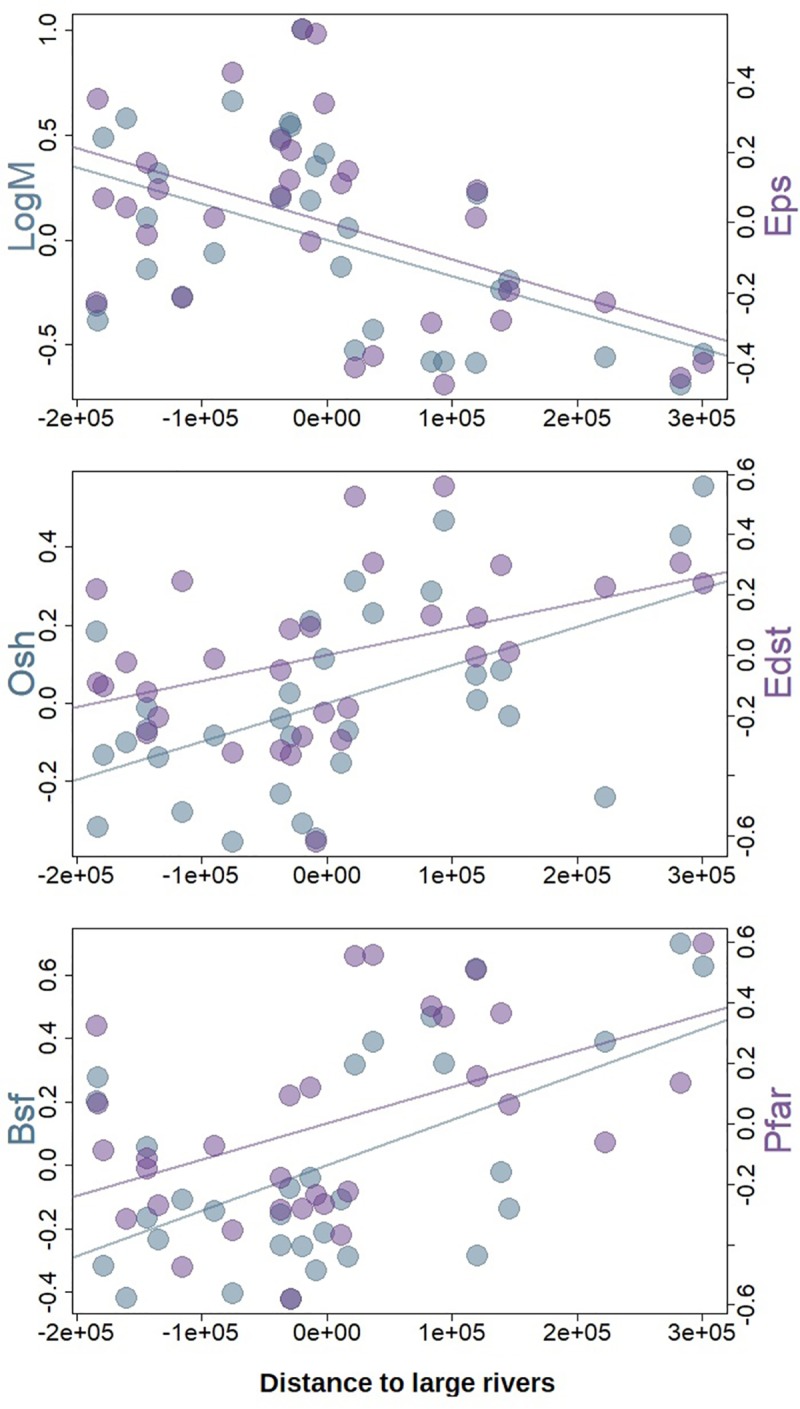
Partial regression derived from the linear mixed-effects models (LMM) investigating the effects of distance to large rivers on CWM with fixed-effect probabilities < 0.05. More details about calculations of functional indices are given in S1 Text. LogM = log (body mass+1); Eps = Eye position; Edst = Eye size; Osh = Oral-gape shape; Bsf = Body transversal surface; PFar = Aspect ratio of the pectoral fin. Distance to the large rivers ranged from 10 to 449 km (See S1 Table for details).

The LMM results showed a significant relationship between the fish functional traits body mass (LogM), body transversal surface (Bsf) and aspect ratio of the pectoral fin (PFar), and distance to large rivers, indicating that assemblages from streams far from large rivers are mainly composed by smaller-sized species with greater swimming ability that inhabit the upper and middle layers of the water column (Fig 5). Furthermore, some traits linked to prey-detection ability (Eps, Edst) and feeding strategy (Osh) were significantly related to distance to large rivers. Stream assemblages closer to large rivers had a higher proportion of species with downturned mouths and low-positioned eyes, indicating an increase in importance of detritivorous habits. There was a higher proportion of species with terminal to superior mouth and laterally positioned eyes (mostly drift feeders on allochthonous food sources) in streams located towards headwaters and far from large rivers. Oral-gape position was the only functional trait significantly related to local abiotic conditions (1^st^ PCA) (Table 2). That axis was mainly related to percentage of coarse litter, mean depth and mean width (more details in S1 Table).

## Discussion

The results corroborated our hypothesis that, at large spatial scales, differences in connection to large rivers are more important than watercourse distance and local conditions in determining functional and taxonomic dissimilarity between stream-fish assemblages. The longitudinal fish zonation that we found is mainly determined by the increase in taxonomic and functional richness close to large rivers and cannot be attributed to an increase in local habitat availability, since all streams are positioned at the headwaters of their respective catchments and are of similar physical dimensions. Similarity within catchments could result in autocorrelation, but the fact that catchments similarly positioned in relation to large rivers, such as catchments 1 and 9, share species and richness patterns is strong evidence of structuring processes unrelated to geographical proximity and the significance values of tests are not affected by autocorrelation, because there was no significant autocorrelation in the residuals of the linear mixed models (S2 Text). This reinforces the hypothesis that stream position in the hydrological network affects the degree of influence of downstream systems on upstream structural patterns through dispersal-mediated interchanges between habitats types, as has been demonstrated in other studies [6,7,10,15,20]. None of the analyzed streams flowed directly into a large river, but the pattern that we found shows that these effects can be detected for many kilometers upstream in the hydrographic catchments that compose a main river basin.

Proximity to floodplains seems to be one of the main factors favoring the constitution of assemblages with higher species richness close to large rivers, since flood pulses allow dispersal that increases the lateral connectivity and the heterogeneity of resources and habitats [20,22,52–54]. Furthermore, most species that inhabit the small streams may be forced to migrate following the retraction of the water level during the dry season, probably using the shallow banks of the larger water courses as refuge areas. These environments are likely to be more available and predictable close to the floodplains and can act as additional habitats for some species, expanding areas for foraging and shelter. These seasonally-available conditions may allow the maintenance of larger local populations and reduce rates of density-dependent mortality [13,54]. Long-term ecological studies in intermittent environments in Central Amazonia are fundamental to better understand the influence of seasonality on local assemblage structure.

Functional changes in fish assemblages along the longitudinal gradient have been frequently reported, mainly related to changes in local conditions [2,5,12,55]. Although large-scale studies are more apt to detect regional than local effects [53], the lack of relationship between the functional patterns and the abiotic conditions in our study is an indication that fish-assemblage structure is not limited by local nutrient dynamics but is also influenced by the ecological processes that occur in surrounding environments [54]. An example is the high frequency of more sedentary and benthic species in stream reaches close to large rivers. These guilds feed on small aquatic invertebrates and/or decomposing material in bottom deposits and are highly dependent on processing of the organic matter in upstream reaches [5,42]. These results indicate that floodplains can buffer the limitation imposed by local conditions and act as a core area for sedentary and benthic species and the accumulated effects of high recruitment of these guilds from the floodplain can be detected at large distances upstream.

Small-sized or miniature species, such as those of the genera *Microcharacium*, *Axelrodia*, *Tythocharax*, *Xenurobrycon*, *Nemuroglanis* and *Priocharax*, were predominant in the assemblages of the most isolated streams. Miniature species rarely live longer than one year in the wild and low nutrient levels in their natural habitats may limit body development of these lineages, which do not exceed 26 mm standard length as adults [56]. The lack of accurate information on the life history of these species makes it difficult to understand their distribution patterns, but it is possible that low dispersal capacity coupled with less nutritional requirements and weaker predation pressure are keeping these species confined to the more isolated headwater drainages [57]. Large-bodied sedentary species, such as members of the genera *Hoplerythrinus*, *Synbranchus*, *Pterygoplichthys*, *Lepthoplosternum* and *Gymnotus*, did not occur in the stream reaches furthest from the large rivers. These species are occasionally caught in low-order streams [20, 42] but do not establish large populations there, only using small streams opportunistically as feeding and shelter areas. These species are likely to be heavily dependent on large water bodies for the maintenance of viable populations.

Our results highlight the importance of connectivity between small catchments and river-floodplain systems for the maintenance of stream-fish assemblage composition, diversity and functional characteristics. The fact that the drainages studied are exposed to seasonal droughts may amplify the role of dispersal in structuring of local assemblages and select species capable of reaching favorable environments throughout the catchment [58–59]. The patterns found show that stream-fish assemblage structure varies according to the drainage position and that generalizations about the ecosystem services provided by fish assemblages in headwaters systems can be inaccurate if they do not incorporate the influence of downstream reaches on upstream assemblages. These findings constitute the baseline to understand the mechanisms that regulate fish movements in the Purus-Madeira interfluve, a threatened region due to the repaving of the BR 319 highway [60]. Future studies of dispersal patterns throughout the catchment and the level of permeability of the landscape to the fish fauna are fundamental to improve our understanding of the probable environmental impacts of road construction on these aquatic ecosystems and to devise effective strategies for protection and monitoring.

## Supporting information

S1 Text Functional-trait measurements. (PDF)

S2 Text Spatial-autocorrelation tests. (PDF)

S1 Table Geographic coordinates of sampled sites and their abiotic variables values. (XLS)

S2 Table List of fish species caught and their functional traits values. More details about functional-trait calculations can be found in S1 Text.
(XLS)
