# Supplementary material for: Distance to large rivers affects fish diversity patterns in highly dynamic streams of Central Amazonia
Source: PLoS One. 2019 Oct 17;14(10):e0223880. doi: 10.1371/journal.pone.0223880 (PMC6797196; doi:10.1371/journal.pone.0223880)
Supplement: S1 Text — (PDF) [file pone.0223880.s001.pdf]

Functional-trait measurements

The functional data used were extracted from an extensive morphometric functional database for Amazonian stream fishes developed by the Igarapés Project. Body mass and morphometric measures were taken on 5 to 12 individuals per species (figure 1) and then combined into ecomorphological traits (Table 1). These traits, except the log-transformed mass, are expressed as unit-less ratios to prevent trivial correlation with body-size. The specimens were weighed using an electronic balance (0.001 g). Body width, mouth width, mouth depth, snout length and protrusion length were measured with a digital caliper (limit of reading 0.1 mm). The other morphological measures were obtained through the use of digital pictures analyzed in Image J software (limit of reading 0.1 mm). We chose 13 functional traits that allowed us to evaluate the functional structure of fish assemblages by characterizing species for three key functions: food acquisition, locomotion, and habitat use.

Figure 1. Fish morphological traits measured from digital pictures

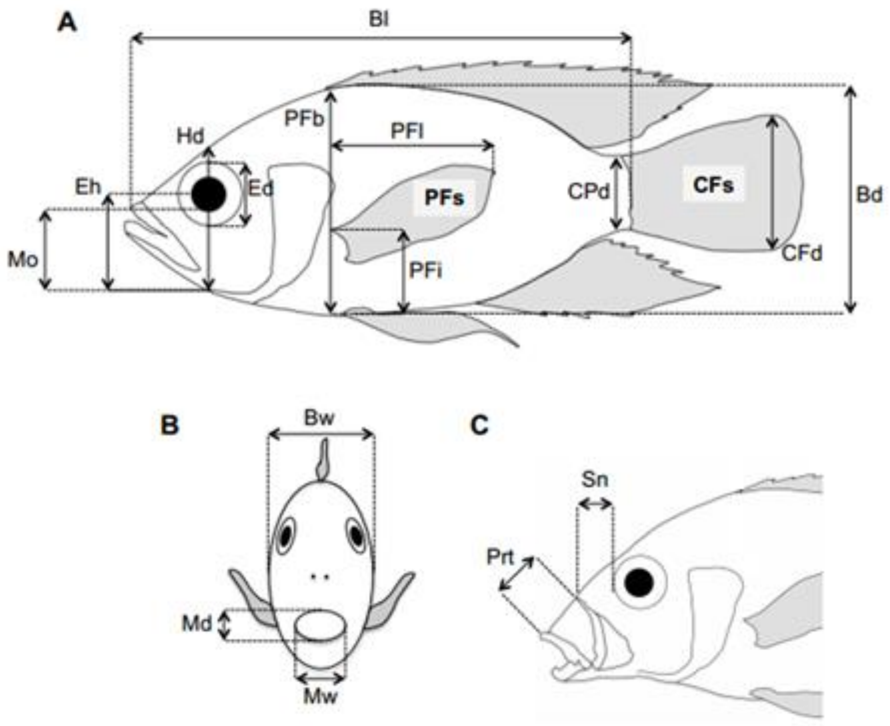

Bd - body depth, CPd - caudal-peduncle minimal depth, CFd - caudal-fin depth, CFs - caudal-fin surface, PFi - distance between the insertion of pectoral fin to the ventral profile, PFb - body depth at

the level of the pectoral-fin insertion, PFl - pectoral-fin length, PFs - pectoral-fin surface, Hd - head depth along the vertical axis of the eye, Ed - eye diameter, Eh - vertical distance between the center of the eye and the ventral profile of the head, Mo - distance from the top of the mouth to the ventral profile of the head along the head depth axis; and with digital caliper (B, C): Bw - body width, Md - mouth depth, Mw - mouth width, Sn - snout length, Prt - protrusion length.

Table 1. List of 13 functional indices calculated from the species ecomorphological traits.

Functional trait	Calculation	Abbreviation	Ecological meaning	Reference
Oral-gape shape	$\frac{Md}{Mw}$	Osh	Way to capture food items	Karpouzi and Stergiou [1]
Oral-gape position	$\frac{Mo}{Hd}$	Ops	Feeding tactic in the water column	Adapted from Sibbing and Nagelkerke [2]
Eye size	$\frac{Ed}{Hd}$	Edst	Prey detection	Adapted from Boyle and Horn [3]
Eye position	$\frac{Eh}{Hd}$	Eps	Vertical position in water column	Gatz [4]
Body transversal shape	$\frac{Bd}{Bw}$	Bsh	Vertical position in water column and hydrodynamism	Sibbing and Nagelkerke [2]
Body transversal surface	$\frac{ln\left[\left(\frac{\pi}{4} * Bw * Bd\right) + 1\right]}{ln(Mass + 1)}$	Bsf	Mass distribution along the body for hydrodynamism	Villéger et al., [5]
Pectoral-fin position	$\frac{PFi}{PFb}$	PFps	Pectoral-fin use for maneuverability	Dumay et al., [6]
Caudal-peduncle throttling	$\frac{CFd}{CPd}$	CPt	Caudal-propulsion efficiency through reduction of drag	Webb [7]

Aspect ratio of the caudal fin	$\frac{CFd^2}{CFs}$	CFar	Caudal-fin use for propulsion and/or direction	Webb [7]
Fin-surface ratio	$\frac{2 * PFs}{CFs}$	Frt	Main type of propulsion between caudal and pectoral fins	Villéger et al., [5]
Fin-surface to body-size ratio	$\frac{(2 * PFs) + CFs}{\frac{\pi}{4} * Bw * Bd}$	Fsf	Acceleration and/or maneuverability efficiency	Villéger et al., [5]
Body mass	$ln(Mass + 1)$	LogM	Metabolism, endurance and swimming ability	Villéger et al., [5]

References of Appendix S1

1. Karpouzi VS, Stergiou KI. The relationships between mouth size and shape and body length for 18 species of marine fishes and their trophic implications. Journal of Fish Biology. 2003; 62:1353-1365.

2. Sibbing FA, Nagelkerke LAJ. Resource partitioning by Lake Tana barbs predicted from fish morphometrics and prey characteristics. Reviews in Fish Biology and Fisheries. 2001;10:393-437.

3. Boyle KS, Horn MH. Comparison of feeding guild structure and ecomorphology of intertidal fish assemblages from central California and central Chile. Marine Ecology Progress Series. 2006; 319:65-84.

4. Gatz AJ. Community organization in fishes as indicated by morphological features. Ecology. 1979; 60: 711 –718.

5. Villéger S, Miranda JR, Hernandez DF, Mouillot, D. Contrasting changes in taxonomic vs. functional diversity of tropical fish communities after habitat degradation. Ecological Application. 2010;20: 1512-1522.

6. Dumay O, Tari PS, Tomasini JA, Mouillot D. Functional groups of lagoon fish species in Languedoc Roussillon, southern France. Journal of Fish Biology. 2004; 64:970-983.

7. Webb PW. Form and function in fish swimming. Scientific American. 1984; 251:72-82.
