# Supplementary material for: Distance to large rivers affects fish diversity patterns in highly dynamic streams of Central Amazonia
Source: PLoS One. 2019 Oct 17;14(10):e0223880. doi: 10.1371/journal.pone.0223880 (PMC6797196; doi:10.1371/journal.pone.0223880)

Support Information Text S2

Table 1. Summary of the partial Mantel correlations test (*r* values/significance values) between taxonomic (A) and functional (B) beta-diversity of stream-fish assemblages and predictor variables, controlling the effects of catchment identity.

	Distance to large rivers difference	Watercourse distance
Tβsør (A)	**0.195/0.015**	0.018/0.424
Tβrich	**0.282/0.002**	**0.154/0.023**
Tβrepl	-0.09/0.883	-0.11/0.95
Fβsør (B)	**0.219/0.015**	0.044/0.321
Fβnes	0.038/0.261	-0.050/0.761
Fβsim	0.119/0.07	0.081/0.168

Figure 1. Moran's coefficients from residuals values of linear mixed-models using taxonomic richness (left) and functional richness (right) as response variable.

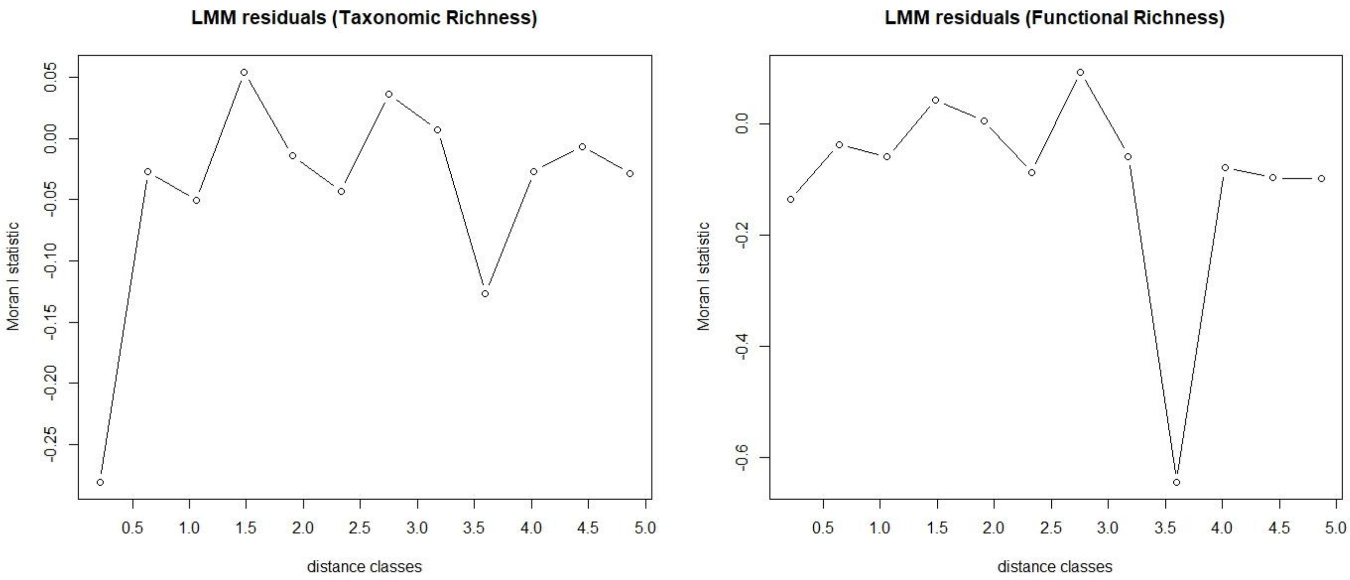

Supplement: S2 Text — (PDF) [file pone.0223880.s002.pdf]
